# Genetic and observational associations of lung function with gastrointestinal tract diseases: pleiotropic and mendelian randomization analysis

**DOI:** 10.1186/s12931-023-02621-0

**Published:** 2023-12-15

**Authors:** Minghui Jiang, Xingjie Hao, Yi Jiang, Si Li, Chaolong Wang, Shanshan Cheng

**Affiliations:** 1 Department of Epidemiology and Biostatistics, School of Public Health, Tongji Medical College, Huazhong University of Science and Technology, Wuhan, 430030 China; 2 Ministry of Education Key Laboratory of Environment and Health, School of Public Health, Tongji Medical College, Huazhong University of Science and Technology, Wuhan, 430030 China

**Keywords:** Gut-lung axis, Lung function, Gastrointestinal tract Disease, Pleiotropic analysis, Mendelian randomization, Epidemiologic observational study

## Abstract

**Background:**

The two-way communications along the gut-lung axis influence the immune function in both gut and lung. However, the shared genetic characteristics of lung function with gastrointestinal tract (GIT) diseases remain to be investigated.

**Methods:**

We first investigated the genetic correlations between three lung function traits and four GIT diseases. Second, we illustrated the genetic overlap by genome-wide pleiotropic analysis (PLACO) and further pinpointed the relevant tissue and cell types by partitioning heritability. Furthermore, we proposed pleiotropic genes as potential drug targets by drug database mining. Finally, we evaluated the causal relationships by epidemiologic observational study and Mendelian randomization (MR) analysis.

**Results:**

We found lung function and GIT diseases were genetically correlated. We identified 258 pleiotropic loci, which were enriched in gut- and lung-specific regions marked by H3K4me1. Among these, 16 pleiotropic genes were targets of drugs, such as tofacitinib and baricitinib targeting *TYK2* for the treatment of ulcer colitis and COVID-19, respectively. We identified a missense variant in *TYK2*, exhibiting a shared causal effect on FEV_1_/FVC and inflammatory bowel disease (rs12720356, *P*_*PLACO*_=1.38 × 10^− 8^). These findings suggested *TYK2* as a promising drug target. Although the epidemiologic observational study suggested the protective role of lung function in the development of GIT diseases, no causalities were found by MR analysis.

**Conclusions:**

Our study suggested the shared genetic characteristics between lung function and GIT diseases. The pleiotropic variants could exert their effects by modulating gene expression marked by histone modifications. Finally, we highlighted the potential of pleiotropic analyses in drug repurposing.

**Supplementary Information:**

The online version contains supplementary material available at 10.1186/s12931-023-02621-0.

## Background

The gut-lung axis represents bidirectional communications between gut and lung and influences the immune function of both organs [1–3]. The comorbidities between chronic lung diseases (such as chronic obstructive pulmonary disease and asthma) and gastrointestinal tract (GIT) diseases (such as inflammatory bowel disease, IBD) have been reported in observational studies [1, 3]. Previous studies emphasized the influence of the microbiota on the gut-lung axis [1, 2], but few studies explored the direct genetic connections between the gut and lung traits [4].

Lung function, as an indicator of lung health, is widely used in the diagnosis and classification of the severity of pulmonary diseases, such as chronic obstructive pulmonary disease [5]. Lung function is usually represented by forced expiratory volume in one second (FEV_1_), forced vital capacity (FVC), and the ratio of FEV_1_ to FVC (FEV_1_/FVC). Genome-wide association studies (GWASs) of lung function have suggested potential biological pathways and drug targets for pulmonary diseases [6, 7]. For instance, Shrine et al. suggested *ITGAV*, a novel genetic signal of FEV_1_/FVC, as a drug target for chronic obstructive pulmonary disease [6]. In addition, GIT diseases are also prevalent and inflict a heavy burden of more than 110 billion dollar cost in 2018 in the United States [8]. The common GIT diseases included peptic ulcer disease (PUD), gastro-oesophageal reflux disease (GORD), IBD, and irritable bowel syndrome (IBS). Previous GWASs have identified many loci associated with GIT diseases [9, 10], such as *FUT2* for PUD, *IL23R* for IBD, and *CADM2* for IBS. Specifically, *IL23R* encodes the interleukin 23 receptor, and risankizumab, targeting interleukin 23, was repurposed as the treatment for Crohn’s disease (a subtype of IBD) from the original usage for psoriasis [11].

Recently, more and more GWAS summary statistics and multi-omics data have become publicly available, for example, the Genotype-Tissue Expression version 8 (GTEx v8) genome and transcriptome data [12]. In addition, many computational approaches have been developed to explore the shared genetic characteristics across traits using only GWAS summary statistics and publicly available omics data, such as genetic correlation estimation, cell type identification, causal inference, and drug repurposing analyses [11, 13–15]. Previous studies have revealed shared genetic characteristics in the gut-brain axis and hepato-ovarian axis by integrated analyses of multi-omics data [16, 17]. However, there are few studies investigating the shared genetic regulatory mechanism between gut-lung axis-related traits. In this study, we proposed to elucidate the genetic overlap and relationships (including correlations and causalities) between traits or diseases in the gut-lung axis and to identify potential drug targets suitable for repurposing by integrating multiple traits and omics data.

## Methods

### GWAS summary statistics

GWAS summary statistics of lung function (FEV_1_, FVC and FEV_1_/FVC, corresponding to GCST007432, GCST007429 and GCST007431) [6] were downloaded from the NHGRI-EBI GWAS catalog [18]. A total of 400,102 Europeans from the UK biobank (UKB) and the SpiroMeta Consortium were analyzed. In each study, residuals of each trait were rank-based inverse normal transformed and used as the phenotype to identify associated variants [6]. For PUD, GORD, and IBD, GWAS summary statistics of 456,327 Europeans from the UKB were downloaded [9]. For IBS, meta-analysis results of 53,400 cases and 433,201 controls from the UKB and the Bellygenes Initiative were downloaded under the study accession GCST90016564 [10] from the NHGRI-EBI GWAS catalog [18]. All summary statistics were in human assembly GRCh37. GIT disease GWASs from the FinnGen (Release 8), which had no sample overlap with the UKB, were used for sensitivity analysis [19]. All GWASs were based on European population to ensure homogeneity of the study population. Details of each GWAS were described in Additional file 1: Table S1.

### Global and local genetic correlation

To identify genetically correlated GIT diseases and lung function trait pairs, the global genetic correlation was assessed using cross-trait linkage disequilibrium score regression (LDSC) [13]. Given the global genetic covariance may be compromised by balanced local genetic covariance (positive genetic covariance partially offsets negative genetic covariance) [20], the local genetic correlation was further quantified using ρ-HESS [20]. For local genetic correlation estimation, the whole genome was partitioned into 1,703 approximately independent linkage disequilibrium (LD) blocks, thus the significant local genetic correlation was identified if the two-tailed *P*-value is less than 2.94 × 10^− 5^ (0.05/1703). The 1,703 local genetic correlations were subsequently added up as the global genetic correlation.

### Pleiotropic analysis to dissect genetic overlap

For each pairwise trait, pleiotropic analysis under composite null hypothesis (PLACO) [21] was used to detect the pleiotropic effect of each variant, where the null hypotheses include $\left\{{\beta }_{1}={\beta }_{2}=0\right\}$, $\left\{{\beta }_{1}=0, {\beta }_{2}\ne 0\right\}$ and $\left\{{\beta }_{1}\ne 0, {\beta }_{2}=0\right\}$, equivalent to testing whether ${\beta }_{1}{\beta }_{2}=0$. Thus, the test statistics of PLACO is ${T}_{PLACO}={Z}_{1}{Z}_{2}$ [21]. The potential pleiotropic variant was identified if *P*_*PLACO*_ is lower than 5 × 10^−8^. According to PLACO recommendation, the variants with minor allele frequency<0.01 or square of Z-scores≥80 were excluded [21].

### Characterization of pleiotropic loci

Clumping implemented in PLINK [22] was used to determine independent loci based on the LD structure of Europeans in the 1000 Genomes Project phase 3 [23]. The variants with LD *r*^*2*^ greater than 0.1 and physical positions within 500 kb from the lead variant were clumped into a locus represented by the lead variant. Nearby loci (distance between LD blocks < 250 kb) were further merged into one genomic locus. The consequence and the nearest gene of each lead variant were annotated using ANNOVAR [24].

### Colocalization analysis

A region spanning a 100 kb window size from each lead variant was chosen to detect whether a causal variant is shared between lung function and GIT disease using coloc [25]. Five hypotheses for pairwise traits in a locus were tested by coloc, including H_0_: no association with either trait; H_1_ or H_2_: association with only trait one or trait two, respectively; H_3_: distinct associations with two traits; H_4_: shared association with both traits. The default arguments were applied with the prior probability of H_1_ or H_2_ as 1 × 10^− 4^, and the prior probability of H_4_ as 1 × 10^− 5^. Then the Bayesian posterior probabilities that integrate all possible configurations were estimated [25]. The pairwise traits were assumed to be colocalized if the posterior probability of H_4_ (PP4) was larger than 0.7 [16].

### Identification of relevant tissue and cell types

Based on pleiotropic results, we estimated the heritability enrichment for each trait pair at 220 tissue and cell-type specific regions marked by histone modifications using stratified LDSC (S-LDSC) [14]. S-LDSC is based on the idea that if a category of SNPs is enriched for heritability then SNPs with high LD to that category will have higher ${\chi }^{2}$ statistics. A total of 220 tissue and cell-type specific annotations were pre-defined based on four histone marks, namely H3K4me1, H3K4me3, H3K9ac, and H3K27ac [14]. For the enrichment testing of each specific annotation, 53 baseline annotations that are not specific to any tissue or cell type were adjusted in the regression model, $E\left({\chi }_{i}^{2}\right)\sim N\sum _{A}{\tau }_{A}l\left(i,A\right)+Nc+1$. $E\left({\chi }_{i}^{2}\right)$ is the expected ${\chi }^{2}$ statistics of SNP $i$; $N$ is the sample size; $A$ represents the annotation categories; ${\tau }_{A}$, the regression coefficient of the category $A$, indicates the per-SNP contribution to heritability of annotation category $A$; $l\left(i,A\right)$ measures the LD scores of SNP $i$ in category $A$; $c$ indicates the contribution of confounding bias. Then a one-sided test (${\tau }_{A}$>0) was conducted to pinpoint the enriched tissue and cell type. The relevant tissue or cell type was identified if the coefficient *P*-value was less than 2.27 × 10^−4^ (0.05/220).

As a sensitivity analysis, MAGMA gene property tests based on gene expression in 54 tissues from the GTEx v8 [12] were conducted using FUMA platform [26]. MAGMA gene property test [27] was based on a linear regression model, $\varvec{Z} \sim {\beta }_{0}+{\varvec{E}}_{\varvec{t}}{\beta }_{t}+{\varvec{E}}_{\varvec{A}}{\beta }_{A}+\varvec{C}{\varvec{\beta }}_{\varvec{C}}+\varvec{\epsilon}$. $\varvec{Z}$ is the gene-based Z-scores calculated from SNP association *P*-values; ${\beta }_{0}$ is the intercept term; ${\varvec{E}}_{\varvec{t}}$ and ${\varvec{E}}_{\varvec{A}}$ are the gene expression of the testing tissue and the average expression of all tissues, respectively, and ${\beta }_{t}$ and ${\beta }_{A}$ are the corresponding effects; $\varvec{C}$ is the confounders, ${\varvec{\beta }}_{\varvec{C}}$ is the effects of confounders, $\varvec{\epsilon}$ is the random errors. A one-sided test (${\beta }_{t}$>0) was performed to identify the positive relationship between gene expression in a specific tissue and the genetic association of genes. The relevant tissue was identified by an association *P*-value less than 9.26 × 10^−4^ (0.05/54). The SNP association *P*-values of PLACO were first integrated into gene-based *P*-values using SNP-wide mean model and the 1000 Genomes Project phase 3 European reference panel, then gene-based *P*-values were converted to the Z-scores [28]; hereafter, the association between the gene-based Z-scores and gene expression in a specific tissue could be investigated by the one-sided test (${\beta }_{t}$>0).

### Drug repurposing

Gene drug interactions were queried in the DrugBank database [29] to identify pleiotropic genes as drug targets. DrugBank is a comprehensive database comprising drug, drug-gene target, drug action, and drug interaction [29]. The latest version 5.1.10 involved over 15,000 drugs, and about 4,000 of them were approved. We focused on 9,344 approved or investigational (in some phase of the drug approval process) drugs.

We performed drug target enrichment analysis to examine whether pleiotropic genes are enriched in genes targeted by drugs in a clinical indication category using GREP [30]. Briefly, two drug databases, DrugBank [29] and Therapeutic Target Database [31], were used to determine drug-target relations. Next, the drugs were categorized by their clinical indication based on two classification systems, namely the Anatomical Therapeutic Chemical (ATC) Classification and the International Classification of Diseases Tenth Revision (ICD-10) curated by the World Health Organization. Subsequently, Fisher’s exact tests were conducted to quantify the enrichment of pleiotropic genes in the drug target of each clinical indication category [30].

### Dissection of causal relationships

The UKB is a population-based longitudinal cohort that collects a wide range of phenotypic and genomic data from more than 500,000 participants [32]. Based on the longitudinal data, the Cox proportional hazard models were applied to identify the effect of each lung function trait on each GIT disease, adjusting for age, sex, and smoking status (ever/never). The definition of the four GIT diseases followed that of GWAS [9]. For example, the definition of IBD included two subtypes, Crohn’s disease and ulcer colitis. For lung function, the best measure of FEV_1_ and FVC from baseline was used. Then, the ratio of FEV_1_ and FVC was calculated. A total of 308,024 Europeans with complete records of GIT disease, lung function, and covariates were kept. Then the incident cases and controls for each trait pair were analyzed. The application number of UKB is 88,159.

Furthermore, bidirectional Mendelian randomization (MR) analyses based on GWAS summary statistics were performed to detect the potential two-way causal relationships between lung function and GIT diseases using the R packages TwoSampleMR [15], mr.raps [33] and Bayesian-weighted MR (BWMR) [34]. A total of 24 trait pairs were tested, thus the statistical significance was defined as *P* < 0.002 (0.05/24) to correct for multiple testing. Inverse-variance weighted method [35] was chosen as the main analysis. Other methods, including MR-Egger [36], weighted median [37], weighted mode [38], BWMR [34], and robust adjusted profile score (RAPS) [33] were applied as sensitivity analyses.

For instrumental variable (IV) selection, independent significant IVs (*P* < 5 × 10^− 8^ with the exposure and LD *r*^*2*^ < 0.01 within 10 Mb based on the 1000 Genomes Project phase 3 Europeans) [23] were kept, while pleiotropic IVs (associated with more than one lung function and GIT traits), IVs with incorrect direction (Steiger test one-sided *P* < 0.05) [39] and MR-PRESSO [40] outliers were removed.

Given the UKB overlapping samples in GWASs for lung function and GIT diseases, summary statistics from the FinnGen [19] for GIT diseases were analyzed in the sensitivity analyses.

## Results

### Overview of the study

**Fig. 1:**
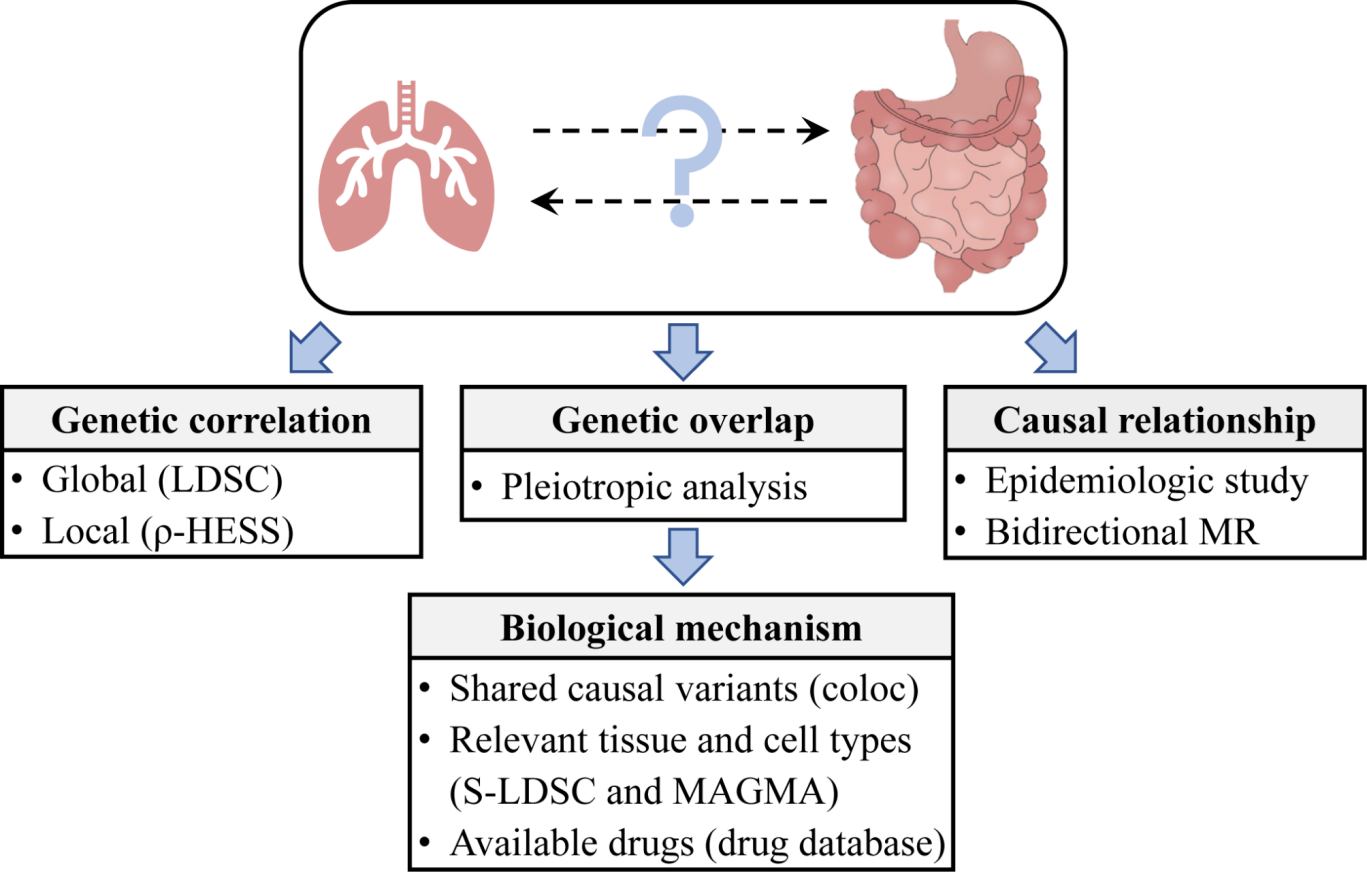
Analyses workflow. To dissect the relationships between lung function and gastrointestinal tract diseases in the gut-lung axis, we first estimated the genetic correlation at both global and local scales. Second, we performed genome-wide pleiotropic analysis to identify shared loci. Subsequently, we deciphered the underlying biological mechanisms by colocalization analysis, S-LDSC, MAGMA gene property analysis, and drug database mining. Third, we examined causal relationships by epidemiologic study and bidirectional MR. LDSC, linkage disequilibrium score regression; S-LDSC, stratified LDSC; MR, Mendelian randomization. The image of gastrointestinal tract was downloaded from https://699pic.com/tupian-401760990.html

We first estimated the genetic correlation between lung function and GIT diseases based on the large-scale GWAS summary statistics from Europeans [6, 9, 10]. We then conducted genome-wide pleiotropic analyses to identify potential shared genetic variants. To further reveal the shared causal variants, we performed colocalization analyses. Based on the pleiotropic results, we performed partitioning heritability and gene-based property analyses to investigate relevant tissue and cell types. Moreover, we searched for the available drugs and conducted drug target enrichment analyses to reveal the potential of pleiotropic genes in drug repurposing. Last, we dissected the causal relationships between lung function and GIT diseases through the Cox proportional hazard models and bidirectional MR analyses. The overall workflow is depicted in Fig. 1.

### Genetic correlations between lung function and gastrointestinal tract diseases

**Fig. 2:**
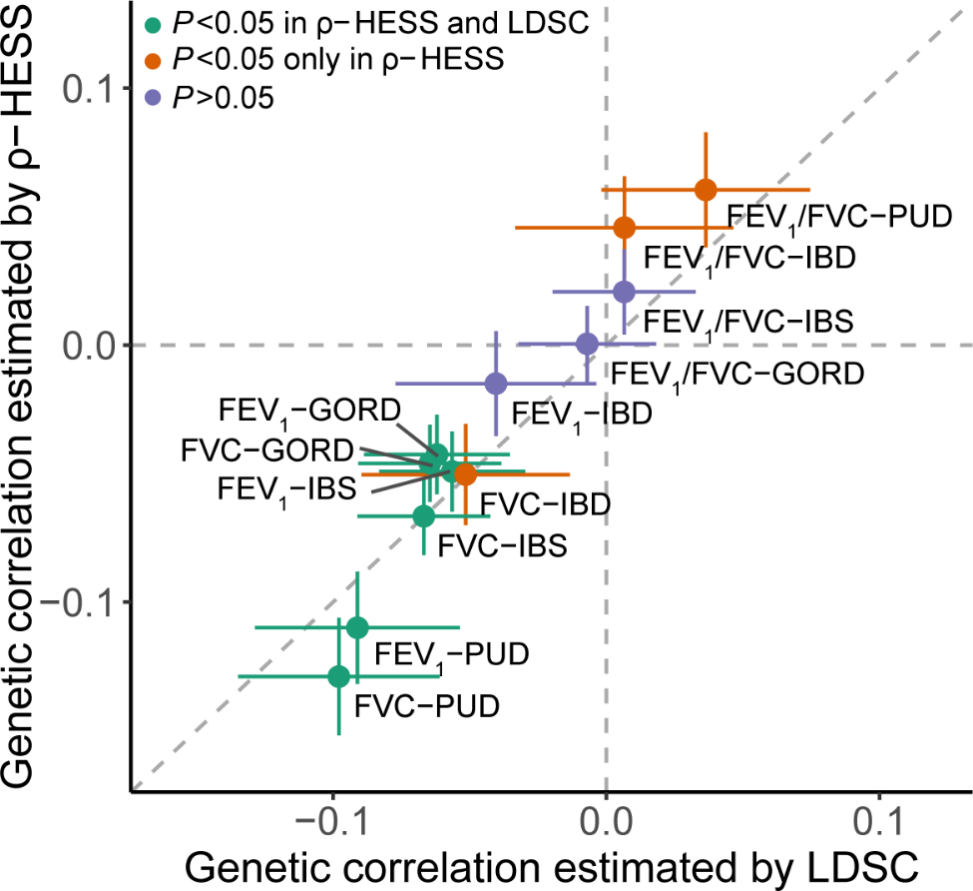
Global genetic correlations estimated by LDSC and ρ-HESS. Genetic correlations between lung function and GIT diseases. Six correlated trait pairs were identified by LDSC and ρ-HESS (green); three correlated trait pairs were identified only by ρ-HESS (orange). Three trait pairs were with *P* > 0.05 (purple). The x- and y-axes represent the estimates of global genetic correlation based on LDSC and ρ-HESS, respectively. The horizontal and vertical dashed lines indicate the genetic correlation is 0; the slope of the diagonal dashed line is 1

The sample size for each GWAS ranged from 400,102 to 486,601 (Additional file 1: Table S1). We found nominally significant global genetic correlations among six trait pairs identified by both LDSC [13] and ρ-HESS [20] (FEV_1_ and FVC with PUD, GORD, and IBS, Fig. 2 and Additional file 1: Table S2). The genetic correlations among six trait pairs were negative and ranged from − 0.129 to − 0.043, indicating GIT diseases were associated with poorer lung function. In addition, another three trait pairs, including FVC-IBD, FEV_1_/FVC-PUD, and FEV_1_/FVC-IBD, were identified by ρ-HESS with genetic correlations as − 0.050, 0.060, and 0.046, respectively (Fig. 2 and Additional file 1: Table S2). Four regions with significant local genetic correlation were identified in FEV_1_-GORD, FEV_1_/FVC-GORD, and FEV_1_/FVC-IBD trait pairs (Additional file 2: Fig. S1 and Additional file 1: Table S3). In summary, ten trait pairs with either statistically significant global or local genetic correlation were identified, including five pairs that passed the multiple testing in the estimation of the global genetic correlation.

### Identification of 258 pleiotropic loci

**Fig. 3:**
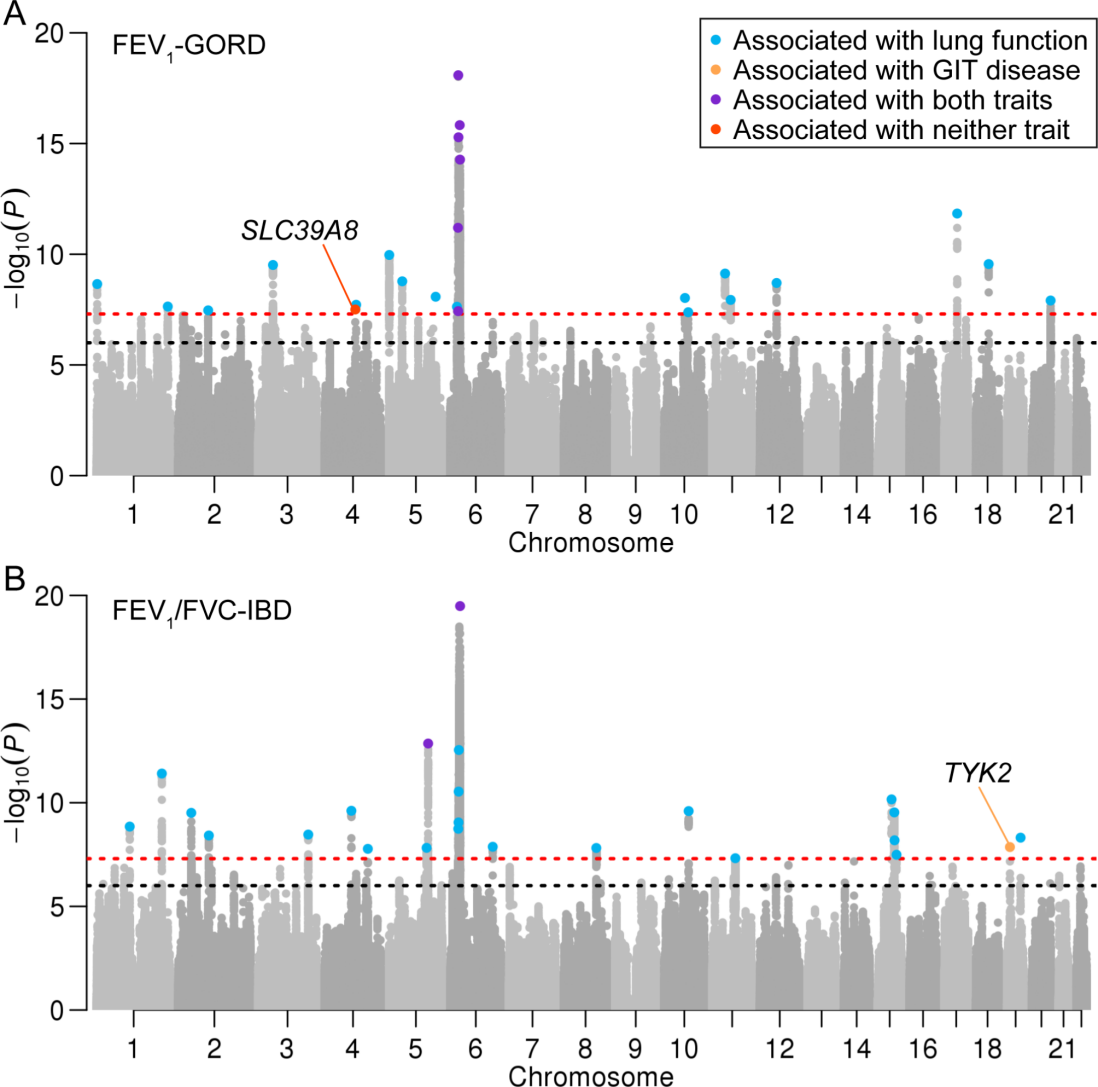
Manhattan plots for the results of pleiotropic analyses. **(A)** FEV_1_-GORD pleiotropic analysis and **(B)** FEV_1_/FVC-IBD pleiotropic analysis. The red dashed lines indicate the genome-wide significance level at *P* = 5 × 10^− 8^, and the black dashed lines indicate the suggestive significance level at *P* = 1 × 10^− 6^. The blue point indicates the locus was associated with lung function (*P*-values of the variants within the lead variant 500 kb were lower than 5$\times$10^−8^); orange indicates the locus was associated with GIT disease; purple indicates the locus was associated with both traits; red indicates the locus was associated with neither trait

We identified 19,058 significant variants, including 10,803 unique variants that showed pleiotropic effects in 12 pairs of lung function and GIT diseases. These variants were further merged into 258 independent genomic loci for pairwise traits, including 227 unique lead variants (Fig. 3, Additional file 2: Fig. S2 and Additional file 1: Table S4). According to the position, 188 unique genes closest to the lead variants were annotated by ANNOVAR [24].

**Fig. 4:**
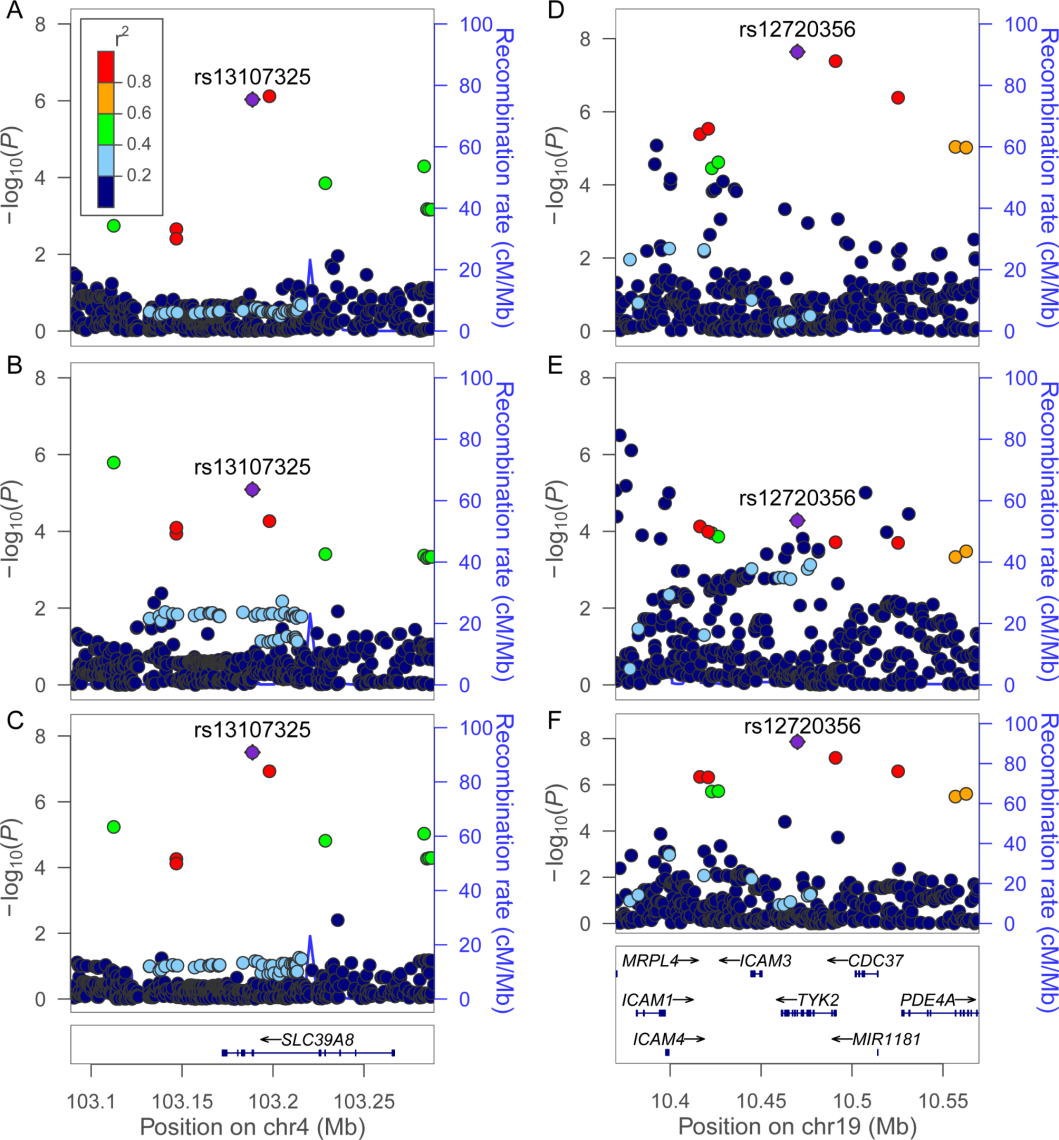
Regional association plots for the pleiotropic loci. (**A-C**) The three panels are GORD GWAS, FEV_1_ GWAS, and pleiotropic analysis, respectively; the colocalization PP4 of GORD and FEV_1_ GWASs was 0.947. (**D-F**) The three panels are IBD GWAS, FEV_1_/FVC GWAS, and pleiotropic analysis, respectively; the colocalization PP4 of IBD and FEV_1_/FVC GWASs was 0.742. The lead variants in the pleiotropic analyses are colored purple, and the other variants are colored based on their LD r^2^ with the lead variant

Specifically, four loci, namely *SLC39A8* in 4q24 for FEV_1_-GORD, *MIR4456* in 5p15.33 for FVC-GORD, *LOC100422212* in 1q23.3 for FEV_1_/FVC-IBS, and *COP1* in 1q25.2 for FEV_1_/FVC-IBS, were not associated with lung function or GIT disease in the original GWASs. The *P*-values of lead variants at these four loci ranged from 1.55 × 10^− 8^ to 4.98 × 10^− 8^ in our pleiotropic analyses, while they ranged from 4.63 × 10^− 7^ to 8.68 × 10^− 6^ in the original GWASs. For instance, we identified a missense variant in *SLC39A8* in the FEV_1_-GORD pleiotropic analysis with lead SNP rs13107325 having *P*_*PLACO*_ = 3.13 × 10^− 8^ (Fig. 3A), but this variant was not associated with FEV_1_ and GORD at the genome-wide significance level (*P*_*FEV1*_=8.17 × 10^− 6^, *P*_*GORD*_=9.30 × 10^− 7^, Fig. 4A-C).

### Colocalization analysis for shared causal variant

For each pleiotropic locus, we performed colocalization analyses to identify the potential causal variant for pairwise traits. Among 258 pleiotropic loci, 59 (22.87%) loci likely had common causal variants for pairwise traits with PP4 > 0.7 and were mapped to 51 unique genes (Fig. 4 and Additional file 1: Table S4). The most potential causal variant (i.e., SNP with the largest PP4) overlapped with the lead variant in 36 pleiotropic loci. Among them, four potential causal variants located in the exon regions: rs4266763 in *SNAPC4* is a synonymous variant, while rs13107325 in *SLC39A8*, rs3197999 in *MST1*, and rs12720356 in *TYK2* are missense variants. In particular, rs13107325 in *SLC39A8* was identified by colocalization analysis for FEV_1_ and GORD (PP4 = 0.827, Fig. 4A-C and Additional file 1: Table S4), as well as for FVC and GORD (PP4 = 0.986, Additional file 1: Table S4).

### Relevant tissue and cell types

**Fig. 5:**
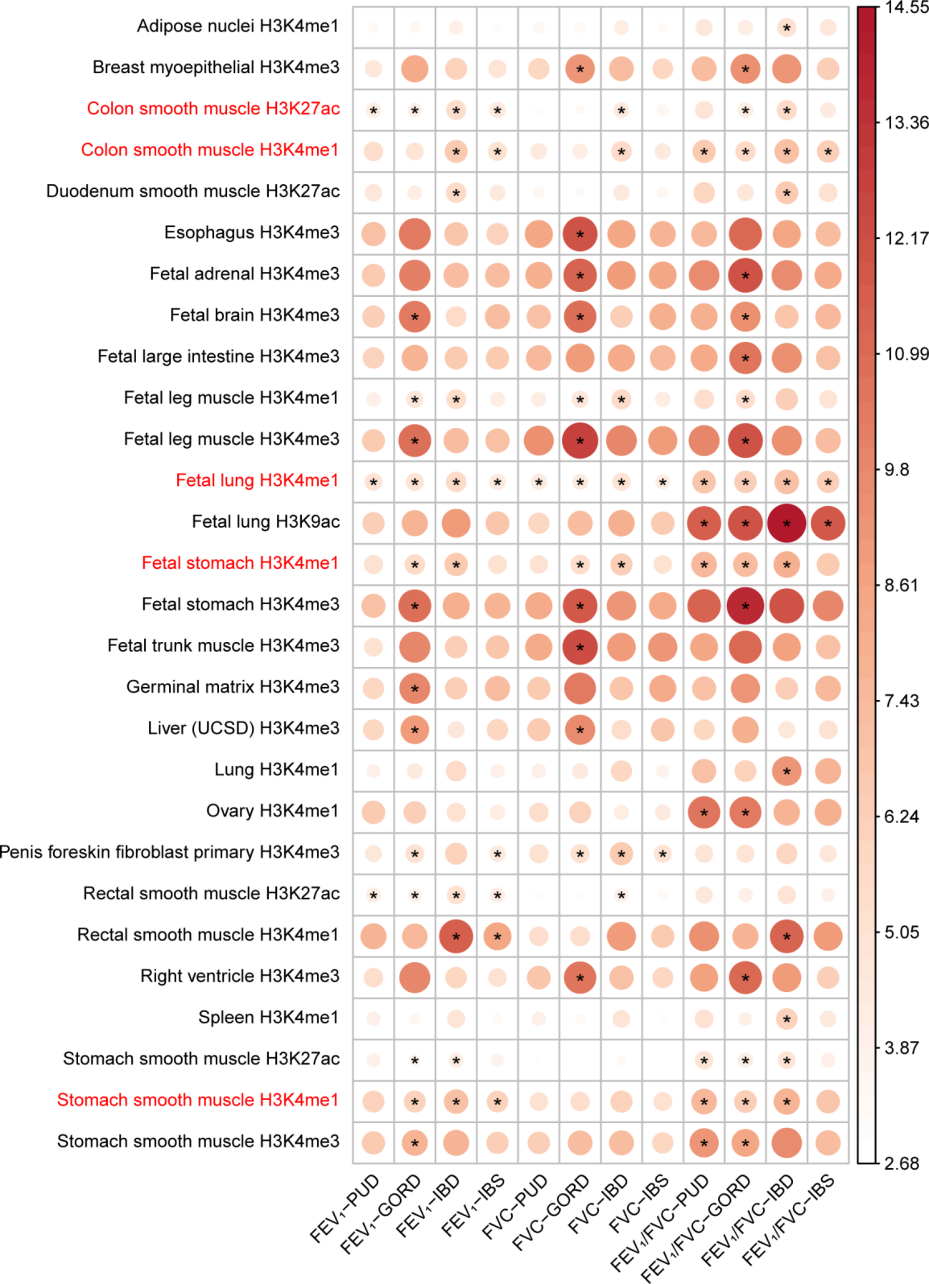
Relevant tissue and cell types for the pleiotropic results. The heritability enrichment for each trait pair was estimated using S-LDSC based on 220 tissue and cell-type specific histone marks. Only the 28 tissue and cell-type specific histone marks that had a *P*-value of less than 0.05/220 in at least one trait pair are presented. Notably, the heritability of a minimum of six trait pairs was found to be enriched in five specific markers, which are highlighted in red. The color and size of the circles indicate the enrichment at the tissue and cell-type specific histone mark. * indicates *P* < 0.05/220

Our findings indicated that the pleiotropic variants were predominantly enriched in 28 specific regions of tissue and cell types, marked by histone modifications. This enrichment was particularly noticeable in the lung and GIT smooth muscle tissues. (Fig. 5). We identified a total of 97 significant associations, of which 45 were associated with the H3K4me1 histone modification (Fig. 5 and Additional file 1: Table S5). Specifically, a minimum of six trait pairs were found to be relevant to several tissues, including colon smooth muscle, fetal lung, fetal stomach, and stomach smooth muscle. In the MAGMA sensitivity analysis, the main positively associated tissues for pleiotropic genes were the GIT and lung tissues, such as the esophagus gastroesophageal junction, colon sigmoid, and lung (Additional file 2: Fig. S3), indicating the pleiotropic genes were enriched in these tissues.

### Drug repurposing analysis

We searched the DrugBank database for available drugs targeting the annotated genes in potential pleiotropic loci [29]. We found 22 pleiotropic loci, which comprised 18 distinct lead variants and were annotated to 16 unique genes, were the targets of approved or investigational drugs (Table 1 and Additional file 1: Table S6). Six drugs in Table 1, namely oxyphencyclimine targeting *CHRM3*, phenethyl isothiocyanate targeting *HSPA4*, crizotinib targeting *MST1R*, pralsetinib targeting *DDR1*, trimebutine targeting *CACNA1D*, and tofacitinib targeting *TYK2*, have been used or studied in trials for the treatment of lung or GIT diseases [29]. For example, oxyphencyclimine is indicated for the treatment of PUD, while crizotinib and pralsetinib are indicated for non-small cell lung cancer [29]. Notably, tofacitinib, the inhibitor drug targeting *TYK2*, is indicated for the treatment of ulcer colitis (a subtype of IBD) [29], while baricitinib, also targeting *TYK2*, is approved for the treatment of COVID-19. In the aforementioned colocalization analysis, we found that a missense variant in *TYK2* was colocalized for FEV_1_/FVC and IBD (rs12720356, *P*_*PLACO*_ = 1.38 × 10^− 8^, PP4 = 0.829, Figs. 3B and 4D-F and Additional file 1: Table S4). The remaining drugs have been used in the treatment of other diseases rather than lung or GIT diseases. For example, fostamatinib, targeting *PIK3C2B*, has been used for the treatment of chronic immune thrombocytopenia; and estramustine, targeting *MAP2*, has been used for prostate cancer [29].

**Table 1:** Pleiotropic genes as targets for approved or investigational drugs

Trait pair	Lead SNP	*P*_*PLACO*_	Nearest Gene	Drug	Indication	Action^*^
FEV_1_-PUD	rs13430465	1.33 × 10^− 8^	*RDH14*	Vitamin A	Vitamin A deficiency	Substrate
FEV_1_-GORD	rs1431721	2.30 × 10^− 8^	*CHRM3*	**Oxyphencyclimine**	Peptic ulcer disease	Antagonist
FEV_1_-IBD	rs1008833	3.26 × 10^− 9^	*PIK3C2B*	Fostamatinib	Chronic immune thrombocytopenia	Inhibitor
FEV_1_-IBD	rs9270979	5.29 × 10^− 24^	*HLA-DRB1*	Glatiramer	Multiple sclerosis	Binder
FEV_1_-IBS	rs4367292	1.23 × 10^− 8^	*HSPA4*	**Phenethyl isothiocyanate**	Leukemia, lung cancer (in trials)	Unknown
FVC-PUD	rs13430465	9.67 × 10^− 9^	*RDH14*	Vitamin A	Vitamin A deficiency	Substrate
FVC-GORD	rs13425141	7.06 × 10^− 9^	*MAP2*	Estramustine	Prostate cancer	Antagonist
FVC-GORD	rs7613360	5.23 × 10^− 10^	*MST1R*	**Crizotinib**	Non-small cell lung cancer	Inhibitor
FVC-IBD	rs1008833	4.91 × 10^− 8^	*PIK3C2B*	Fostamatinib	Chronic immune thrombocytopenia	Inhibitor
FVC-IBD	rs4705885	7.98 × 10^− 11^	*HINT1*	Sofosbuvir	Hepatitis C virus infections	Substrate
FVC-IBD	rs9270911	1.16 × 10^− 23^	*HLA-DRB1*	Glatiramer	Multiple sclerosis	Binder
FVC-IBS	rs9268846	2.16 × 10^− 9^	*HLA-DRA*	1D09C3	Cancer (in trials)	Unknown
FVC-IBS	rs3017666	2.28 × 10^− 11^	*GANAB*	Miglitol	Non-insulin-dependent diabetes mellitus	Antagonist
FEV_1_/FVC-PUD	rs1264318	1.13 × 10^− 9^	*DDR1*	**Pralsetinib**	Non-small cell lung cancer	Inhibitor
FEV_1_/FVC-GORD	rs11720018	9.10 × 10^− 10^	*CACNA1D*	**Trimebutine**	Irritable bowel syndrome	Inhibitor
FEV_1_/FVC-GORD	rs4790311	1.10 × 10^− 8^	*SMG6*	Grn163l	Leukemia and solid tumors	Unknown
FEV_1_/FVC-IBD	rs9270911	3.28 × 10^− 20^	*HLA-DRB1*	Glatiramer	Multiple sclerosis	Binder
FEV_1_/FVC-IBD	rs4944210	4.76 × 10^− 8^	*NARS2*	Asparagine	Nutritional supplementation	Unknown
FEV_1_/FVC-IBD	rs12720356	1.38 × 10^− 8^	*TYK2*	**Tofacitinib**	Ulcerative colitis	Inhibitor
FEV_1_/FVC-IBS	rs4367292	2.50 × 10^− 8^	*HSPA4*	**Phenethyl isothiocyanate**	Leukemia, lung cancer (in trials)	Unknown
FEV_1_/FVC-IBS	rs9260603	3.05 × 10^− 13^	*HLA-A*	Nelipepimut-S	Prostate and breast cancer.	Unknown
FEV_1_/FVC-IBS	rs8070954	3.63 × 10^− 8^	*SMG6*	Grn163l	Leukemia and solid tumors	Unknown

For each gene, only one available drug was listed (all drugs are listed in Additional file 1: Table S6). Drugs indicated for gastrointestinal tract and lung diseases were marked in bold. ^*^: Unknown indicates the drug belongs to the investigational group in the DrugBank database

In the drug target enrichment analysis, we found the 188 pleiotropic genes were enriched in the target of drugs for functional gastrointestinal disorders (*P* = 0.018) and symptoms and signs involving the digestive system and abdomen (*P* = 0.036) (Additional file 1: Table S7). The nominally significant enrichment implied that the pleiotropic genes were likely suitable for drug repurposing in GIT diseases.

### Causal inference between lung function and gastrointestinal tract diseases

We first used Cox proportional hazard models to identify the effect of lung function on GIT diseases in the UKB cohort. The number of incident cases for each GIT disease ranged from 1,756 to 24,182 (Additional file 2: Fig. S4). We found that higher FEV_1_ or FVC was protective against GIT disease (Additional file 2: Fig. S4). The hazard ratios (HR) with the 95% confidence interval (CI) of FEV_1_ on PUD, GORD, IBD, and IBS were 0.74 (0.70, 0.77), 0.86 (0.84, 0.88), 0.78 (0.72, 0.85), and 0.83 (0.79, 0.88), respectively. The HR (95% CI) of FVC on the four GIT diseases were 0.80 (0.77, 0.83), 0.87 (0.86, 0.89), 0.84 (0.79, 0.90), and 0.85 (0.81, 0.88), respectively. All the *P*-values were less than 8 × 10^− 7^. The relationships between FEV_1_/FVC and GIT diseases were complicated, with protective effects on PUD and IBD, while no effect on GORD and IBS (*P* > 0.05).

Next, bidirectional MR analyses were performed to detect the two-way causal relationships between three lung function traits and four GIT diseases. When IBS was the exposure and FEV_1_/FVC was the outcome, we only identified one valid instrumental variable (IV) after MR-PRESSO outlier exclusion, thus the main MR analysis was not applicable. For the other pairs of traits, no statistically significant causal effect was detected after the Bonferroni correction, although PUD showed a nominal positive effect on FVC (*P* = 0.003), and FEV_1_/FVC was positively associated with IBS (*P* = 0.028) (Additional file 2: Fig. S5, Additional file 1: Tables S8 and S9). Similarly, we found no significant causal effect in the sensitivity analyses when the GWASs of GIT diseases were from the FinnGen study and thus there was no sample overlap in the GWASs for exposures and outcomes (Additional file 1: Tables S10 and S11).

## Discussion

In this study, we explored the shared genetic effects in the gut-lung axis traits and diseases. We found that lung function was genetically correlated with GIT diseases, while they showed no causal relationships with each other. Based on pleiotropic analyses, we revealed significant genetic overlap and relevant tissues. Furthermore, some potential drugs for repurposing were suggested for the treatment of lung function and GIT diseases.

We observed negative genetic correlations in the pairwise FEV_1_-GIT and FVC-GIT diseases, probably reflecting the genetic risks of GIT diseases related to lower FEV_1_ and FVC, while the positive correlations in FEV_1_/FVC-PUD and FEV_1_/FVC-IBD trait pairs might reflect the genetic risks of GIT diseases related to more degree of decreased FVC than FEV_1_. However, the estimated global genetic correlations in FEV_1_-GORD, FVC-IBD, FEV_1_/FVC-PUD, and FEV_1_/FVC-IBD trait pairs were not significant after the multiple testing correction (*P* = 0.006, 0.011, 0.007, and 0.022 in ρ-HESS, respectively), probably due to insufficient power caused by the small number of cases of the GIT disease GWASs, such as the 7,045 and 16,666 cases in the IBD and PUD GWASs, respectively. Besides, the bidirectional genetic covariance among different genomic regions might neutralize the global genetic correlation estimates, which highlights the importance of the estimation of the local genetic correlation [20]. For example, we observed a significant local genetic correlation (*r*_g_=0.586, *P* = 2.39 × 10^− 5^) in 6p21.33 between FEV_1_/FVC and GORD, while the global correlation was not significant. Although the local genetic correlations covered the genomic regions spanning about 1.6 Mb in width on average [20], the resolution is still not high enough to neglect the influence of the heterogeneous effects on the estimation. For instance, we observed inconsistent effects across variants in the FEV_1_-GORD pleiotropic analysis, specifically 14 of the 24 lead variants showed the same effect direction between FEV_1_ and GORD GWASs, while the other ten lead variants showed the reverse effect direction. Thus, the pleiotropic analysis of a single variant provided finer granularity to explore the shared genetic characteristics across traits.

The pleiotropic analyses showed significant genetic overlap between lung function and GIT diseases. We identified a missense variant in *SLC39A8* (rs13107325, *P*_*PLACO*_=3.13 × 10^− 8^ for FEV_1_-GORD and *P*_*PLACO*_=5.72 × 10^− 11^ for FVC-GORD). The two pairs of traits were both colocalized in *SLC39A8* with PP4 greater than 0.9. *SLC39A8* (solute carrier family 39 member 8) encodes a member of zinc transporter proteins and functions in the import of zinc from extracellular and intracellular areas to the cytoplasm. Zinc homeostasis is crucial for immune function which plays an important role in inflammation [41]. Given that inflammation can lead to lung function impairment [42], and inflammation in the esophagus is a complication of GORD [43], we postulate that rs13107325 might affect lung function and the progression of GORD through the dysfunction of zinc transportation and subsequent immune imbalance.

In addition, we identified a missense variant in *TYK2*, associated with FEV_1_/FVC and IBD (rs12720356, *P*_*PLACO*_=1.38 × 10^− 8^). FEV_1_/FVC and IBD were colocalized with PP4 of 0.742. *TYK2* (tyrosine kinase 2) encodes a member of the tyrosine kinase and functions in signal transduction of diverse cytokines, such as interleukin 12 and type I interferons, which can further regulate the inflammatory process [44]. Notably, *TYK2* inhibition has been established as a promising therapeutic target for immune-mediated inflammatory diseases [44]. Tofacitinib, an inhibitor of *TYK2*, has been used in the treatment of ulcerative colitis, and baricitinib has been approved for the treatment of COVID-19 [29]. Based on the pleiotropy and colocalization results, *TYK2* was likely a promising drug target for both lung and GIT diseases.

We identified other pleiotropic genes involved in the immune response. For instance, *MST1* (macrophage stimulating 1, encoding a growth factor protein produced by macrophages) and its receptor *MST1R* have been shown to play an important role in immune regulation and inflammation response [45, 46]. We found that *MST1* showed pleiotropic effects in FEV_1_-IBD and FVC-IBD pairwise traits with the lead variant rs3197999 (a missense variant, *P*_*PLACO*_=1.73 × 10^− 11^ and 1.53 × 10^− 10^, respectively). rs3197999 was a significant eQTL for *MST1* in GTEx v8 multiple tissues, with *P* = 5.5 × 10^− 16^ in esophagus mucosa [12]. Additionally, a shared variant near *MST1R* was identified in FVC-GORD pleiotropic analysis (*P*_*PLACO*_=5.23 × 10^− 10^). Notably, crizotinib, the inhibitory drug of *MST1R*, has been used to treat non-small cell lung cancer. These findings highlighted the drug-repurposing potential of immune-related genes for lung and GIT diseases.

Furthermore, we found other pleiotropic genes as drug targets for lung and GIT diseases, such as pralsetinib targeting *DDR1* for the treatment of non-small cell lung cancer and trimebutine targeting *CACNA1D* for the treatment of IBS. We also observed nominally significant enrichment of pleiotropic genes in drug targets indicated for digestive system diseases. These findings emphasized pleiotropic genes as targets for drug repurposing. The proposed repurposed drugs were based on drug database mining and reflected the observational effects. Thus, future clinical studies are required to investigate whether these drugs are effective in the treatment of lung and GIT diseases.

To reveal the shared biological mechanism, we performed the S-LDSC and MAGMA gene property analyses. We observed that pleiotropic variants were relevant to GIT and lung tissues by both methods. For example, we found *SLC39A8* was highly expressed in the GTEx v8 lung tissue, and *TYK2* was ubiquitously expressed in lung, colon, and esophagus tissues (Additional file 2: Fig. S6) [12]. Furthermore, *SLC39A8* expression was specifically enhanced in lung and alveolar cells [47, 48]. Moreover, we discovered that variants exhibiting pleiotropy were most significantly enriched in tissue and cell type-specific regions marked by H3K4me1. This suggests that these pleiotropic variants may exert their effects by regulating gene expression within these specific tissues. These findings highlighted the shared biological mechanism between lung function and GIT diseases.

Although we observed the protective effect of lung function on GIT disease in the epidemiologic study, we did not observe significant causal relationships between lung function and GIT diseases in the bidirectional MR analyses. Given that epidemiologic studies may be affected by undetected confounding, while MR is less susceptible to confounding effects, we suggested that GIT diseases and lung function are more likely to be associated rather than causative. The pleiotropic genes might influence pairwise traits through horizontal pleiotropy, or other ways such as gut microbes, rather than vertical pleiotropy (causality). Horizontal pleiotropic genes might facilitate drug repurposing because the drug targets could influence both traits simultaneously.

There were several limitations in our study. First, due to the relatively small number of cases of GIT diseases, the statistical power might be insufficient, especially for the global genetic correlation estimation. Therefore, we used the nominal significance threshold and further focused on pleiotropic analyses of each variant to determine the shared genetic characteristics across traits. Second, there were overlapped samples between lung function and GIT disease GWASs, which may bias the causal estimates of two-sample MR. To address this concern, we further performed MR sensitivity analyses based on GIT GWAS summary statistics from the FinnGen to avoid sample overlap with the UKB. Third, although we identified pleiotropic variants present in lung function and GIT diseases, we did not observe causal relationships between lung function and GIT diseases, suggesting the complex genetic (including both global and local) and phenotypic relationships underlying lung and gut diseases. Fourth, we focused exclusively on the European population, which attenuated the bias of population structure but restricted the application of our findings to other populations. Replication in other populations is needed. Fifth, we investigated the relationships between GIT diseases and lung function, instead of pulmonary diseases, which may neglect the direct associations between lung and gut diseases. Further studies that focus on the shared genetic characteristics of pulmonary diseases with GIT diseases are needed. However, it is important to note that lung function serves as a crucial indicator of lung health. Emphasizing lung function can aid in the early detection of changes related to pulmonary diseases and support the development of effective therapeutic approaches.

## Conclusions

In conclusion, our study revealed the genetic correlations and genetic overlap, but not causal relationships between lung function and GIT diseases. The pleiotropic genes could be used as drug targets of lung and GIT diseases and were enriched in drug targets indicated for digestive system diseases, highlighting their potential in drug repurposing.

### Electronic supplementary material

Below is the link to the electronic supplementary material.

Supplementary Material 1

Supplementary Material 2

## Data Availability

The datasets analyzed during the current study are available at the Program in Complex Trait Genomics (https://cnsgenomics.com/content/data), GWAS catalog (https://www.ebi.ac.uk/gwas/), and FinnGen (https://www.finngen.fi/en/access_results) with the PMID listed in Additional file 1: Table S1.

## Abbreviations

ATC: Anatomical Therapeutic Chemical
FEV_1_: Forced expiratory volume in one second
FEV_1_/FVC: The ratio of FEV_1_ to FVC
FVC: Forced vital capacity
GIT: Gastrointestinal tract
GORD: Gastro-oesophageal reflux disease
GTEx v8: Genotype-Tissue Expression version 8
GWASs: Genome-wide association studies
IBD: Inflammatory bowel disease
IBS: Irritable bowel syndrome
ICD-10: International Classification of Diseases Tenth Revision
IV: Instrumental variable
LD: Linkage disequilibrium
LDSC: Linkage disequilibrium score regression
MR: Mendelian randomization
PLACO: Pleiotropic analysis under composite null hypothesis
PP4: Posterior probability of H_4_
PUD: Peptic ulcer disease
S-LDSC: Stratified LDSC
UKB: UK biobank

## References

[1] Budden KF, Gellatly SL, Wood DL, Cooper MA, Morrison M, Hugenholtz P, Hansbro PM (2017). Emerging pathogenic links between microbiota and the gut-lung axis. Nat Rev Microbiol.

[2] Wypych TP, Wickramasinghe LC, Marsland BJ (2019). The influence of the microbiome on respiratory health. Nat Immunol.

[3] Wang L, Cai Y, Garssen J, Henricks PAJ, Folkerts G, Braber S (2023). The bidirectional gut-lung Axis in Chronic Obstructive Pulmonary Disease. Am J Respir Crit Care Med.

[4] Keely S, Talley NJ, Hansbro PM (2012). Pulmonary-intestinal cross-talk in mucosal inflammatory Disease. Mucosal Immunol.

[5] Vogelmeier CF, Criner GJ, Martinez FJ, Anzueto A, Barnes PJ, Bourbeau J, Celli BR, Chen R, Decramer M, Fabbri LM (2017). Global strategy for the diagnosis, management, and Prevention of Chronic Obstructive Lung Disease 2017 Report. GOLD Executive Summary. Am J Respir Crit Care Med.

[6] Shrine N, Guyatt AL, Erzurumluoglu AM, Jackson VE, Hobbs BD, Melbourne CA, Batini C, Fawcett KA, Song K, Sakornsakolpat P (2019). New genetic signals for lung function highlight pathways and Chronic Obstructive Pulmonary Disease associations across multiple ancestries. Nat Genet.

[7] Wyss AB, Sofer T, Lee MK, Terzikhan N, Nguyen JN, Lahousse L, Latourelle JC, Smith AV, Bartz TM, Feitosa MF (2018). Multiethnic meta-analysis identifies ancestry-specific and cross-ancestry loci for pulmonary function. Nat Commun.

[8] Peery AF, Crockett SD, Murphy CC, Jensen ET, Kim HP, Egberg MD, Lund JL, Moon AM, Pate V, Barnes EL (2022). Burden and cost of gastrointestinal, liver, and pancreatic Diseases in the United States: Update 2021. Gastroenterology.

[9] Wu Y, Murray GK, Byrne EM, Sidorenko J, Visscher PM, Wray NR (2021). GWAS of Peptic Ulcer Disease implicates Helicobacter pylori Infection, other gastrointestinal disorders and depression. Nat Commun.

[10] Eijsbouts C, Zheng T, Kennedy NA, Bonfiglio F, Anderson CA, Moutsianas L, Holliday J, Shi J, Shringarpure S, Agee M (2021). Genome-wide analysis of 53,400 people with irritable bowel syndrome highlights shared genetic pathways with mood and anxiety disorders. Nat Genet.

[11] Reay WR, Cairns MJ (2021). Advancing the use of genome-wide association studies for drug repurposing. Nat Rev Genet.

[12] Consortium GT (2020). The GTEx Consortium atlas of genetic regulatory effects across human tissues. Science.

[13] Bulik-Sullivan B, Finucane HK, Anttila V, Gusev A, Day FR, Loh PR, ReproGen C, Psychiatric Genomics C, Control C, Duncan L, Genetic Consortium for Anorexia Nervosa of the Wellcome Trust Case (2015). An atlas of genetic correlations across human Diseases and traits. Nat Genet.

[14] Finucane HK, Bulik-Sullivan B, Gusev A, Trynka G, Reshef Y, Loh PR, Anttila V, Xu H, Zang C, Farh K (2015). Partitioning heritability by functional annotation using genome-wide association summary statistics. Nat Genet.

[15] Hemani G, Zheng J, Elsworth B, Wade KH, Haberland V, Baird D, Laurin C, Burgess S, Bowden J, Langdon R et al. The MR-Base platform supports systematic causal inference across the human phenome. Elife 2018, 7. 10.7554/eLife.34408 PMC5976434 29846171

[16] Gong W, Guo P, Li Y, Liu L, Yan R, Liu S, Wang S, Xue F, Zhou X, Yuan Z. Role of the gut-brain Axis in the Shared Genetic Etiology between Gastrointestinal Tract Diseases and Psychiatric disorders. JAMA Psychiatry 2023, 80. 10.1001/jamapsychiatry.2022.4974 PMC9909581 36753304

[17] Liu D, Gao X, Pan XF, Zhou T, Zhu CR, Li F, Fan JG, Targher G, Zhao J. The hepato-ovarian axis: genetic evidence for a causal association between non-alcoholic fatty Liver Disease and polycystic ovary syndrome. BMC Med 2023, 21. 10.1186/s12916-023-02775-0 PMC9940436 36800955

[18] MacArthur J, Bowler E, Cerezo M, Gil L, Hall P, Hastings E, Junkins H, McMahon A, Milano A, Morales J (2017). The new NHGRI-EBI catalog of published genome-wide association studies (GWAS catalog). Nucleic Acids Res.

[19] Kurki MI, Karjalainen J, Palta P, Sipila TP, Kristiansson K, Donner KM, Reeve MP, Laivuori H, Aavikko M, Kaunisto MA (2023). FinnGen provides genetic insights from a well-phenotyped isolated population. Nature.

[20] Shi H, Mancuso N, Spendlove S, Pasaniuc B (2017). Local genetic correlation gives insights into the Shared Genetic Architecture of Complex traits. Am J Hum Genet.

[21] Ray D, Chatterjee N (2020). A powerful method for pleiotropic analysis under composite null hypothesis identifies novel shared loci between type 2 Diabetes and Prostate Cancer. PLoS Genet.

[22] Purcell S, Neale B, Todd-Brown K, Thomas L, Ferreira MA, Bender D, Maller J, Sklar P, de Bakker PI, Daly MJ, Sham PC (2007). PLINK: a tool set for whole-genome association and population-based linkage analyses. Am J Hum Genet.

[23] Genomes Project C, Auton A, Brooks LD, Durbin RM, Garrison EP, Kang HM, Korbel JO, Marchini JL, McCarthy S, McVean GA, Abecasis GR (2015). A global reference for human genetic variation. Nature.

[24] Wang K, Li M, Hakonarson H (2010). ANNOVAR: functional annotation of genetic variants from high-throughput sequencing data. Nucleic Acids Res.

[25] Giambartolomei C, Vukcevic D, Schadt EE, Franke L, Hingorani AD, Wallace C, Plagnol V (2014). Bayesian test for colocalisation between pairs of genetic association studies using summary statistics. PLoS Genet.

[26] Watanabe K, Taskesen E, van Bochoven A, Posthuma D (2017). Functional mapping and annotation of genetic associations with FUMA. Nat Commun.

[27] de Leeuw CA, Stringer S, Dekkers IA, Heskes T, Posthuma D (2018). Conditional and interaction gene-set analysis reveals novel functional pathways for blood pressure. Nat Commun.

[28] de Leeuw CA, Mooij JM, Heskes T, Posthuma D (2015). MAGMA: generalized gene-set analysis of GWAS data. PLoS Comput Biol.

[29] Wishart DS, Feunang YD, Guo AC, Lo EJ, Marcu A, Grant JR, Sajed T, Johnson D, Li C, Sayeeda Z (2018). DrugBank 5.0: a major update to the DrugBank database for 2018. Nucleic Acids Res.

[30] Sakaue S, Okada Y (2019). GREP: genome for REPositioning Drugs. Bioinformatics.

[31] Li YH, Yu CY, Li XX, Zhang P, Tang J, Yang Q, Fu T, Zhang X, Cui X, Tu G (2018). Therapeutic target database update 2018: enriched resource for facilitating bench-to-clinic research of targeted therapeutics. Nucleic Acids Res.

[32] Bycroft C, Freeman C, Petkova D, Band G, Elliott LT, Sharp K, Motyer A, Vukcevic D, Delaneau O, O’Connell J (2018). The UK Biobank resource with deep phenotyping and genomic data. Nature.

[33] Zhao Q, Wang J, Hemani G, Bowden J, Small DS (2020). Statistical inference in two-sample summary-data mendelian randomization using robust adjusted profile score. Ann Stat.

[34] Zhao J, Ming J, Hu X, Chen G, Liu J, Yang C (2020). Bayesian weighted mendelian randomization for causal inference based on summary statistics. Bioinformatics.

[35] Burgess S, Butterworth A, Thompson SG (2013). Mendelian randomization analysis with multiple genetic variants using summarized data. Genet Epidemiol.

[36] Bowden J, Davey Smith G, Burgess S (2015). Mendelian randomization with invalid instruments: effect estimation and bias detection through Egger regression. Int J Epidemiol.

[37] Bowden J, Davey Smith G, Haycock PC, Burgess S (2016). Consistent estimation in mendelian randomization with some Invalid instruments using a weighted median estimator. Genet Epidemiol.

[38] Hartwig FP, Davey Smith G, Bowden J (2017). Robust inference in summary data mendelian randomization via the zero modal pleiotropy assumption. Int J Epidemiol.

[39] Hemani G, Tilling K, Davey Smith G (2017). Orienting the causal relationship between imprecisely measured traits using GWAS summary data. PLoS Genet.

[40] Verbanck M, Chen CY, Neale B, Do R (2018). Detection of widespread horizontal pleiotropy in causal relationships inferred from mendelian randomization between complex traits and Diseases. Nat Genet.

[41] Wessels I, Maywald M, Rink L. Zinc as a gatekeeper of Immune function. Nutrients 2017, 9. 10.3390/nu9121286 PMC5748737 29186856

[42] Baines KJ, Backer V, Gibson PG, Powel H, Porsbjerg CM (2015). Impaired lung function is associated with systemic inflammation and macrophage activation. Eur Respir J.

[43] Clarrett DM, Hachem C (2018). Gastroesophageal reflux Disease (GERD). Mo Med.

[44] Rusinol L, Puig L. Tyk2 targeting in Immune-mediated inflammatory Diseases. Int J Mol Sci 2023, 24. 10.3390/ijms24043391 PMC9959504 36834806

[45] Qin F, Tian J, Zhou D, Chen L (2013). Mst1 and Mst2 kinases: regulations and Diseases. Cell Biosci.

[46] Tian Y, Song H, Jin D, Hu N, Sun L (2020). MST1-Hippo pathway regulates inflammation response following Myocardial Infarction through inhibiting HO-1 signaling pathway. J Recept Signal Transduct Res.

[47] Uhlén M, Fagerberg L, Hallström BM, Lindskog C, Oksvold P, Mardinoglu A, Sivertsson Å, Kampf C, Sjöstedt E, Asplund A et al. Tissue-based map of the human proteome. Science 2015, 347. 10.1126/science.1260419 25613900

[48] Karlsson M, Zhang C, Méar L, Zhong W, Digre A, Katona B, Sjöstedt E, Butler L, Odeberg J, Dusart P et al. A single-cell type transcriptomics map of human tissues. Sci Adv 2021, 7. 10.1126/sciadv.abh2169 PMC8318366 34321199

