# Supplementary material for: Genetic and observational associations of lung function with gastrointestinal tract diseases: pleiotropic and mendelian randomization analysis
Source: Respir Res. 2023 Dec 15;24:315. doi: 10.1186/s12931-023-02621-0 (PMC10724909; doi:10.1186/s12931-023-02621-0)
Supplement: Supplementary file 2 — Supplementary Material 2 [file 12931_2023_2621_MOESM2_ESM.docx]

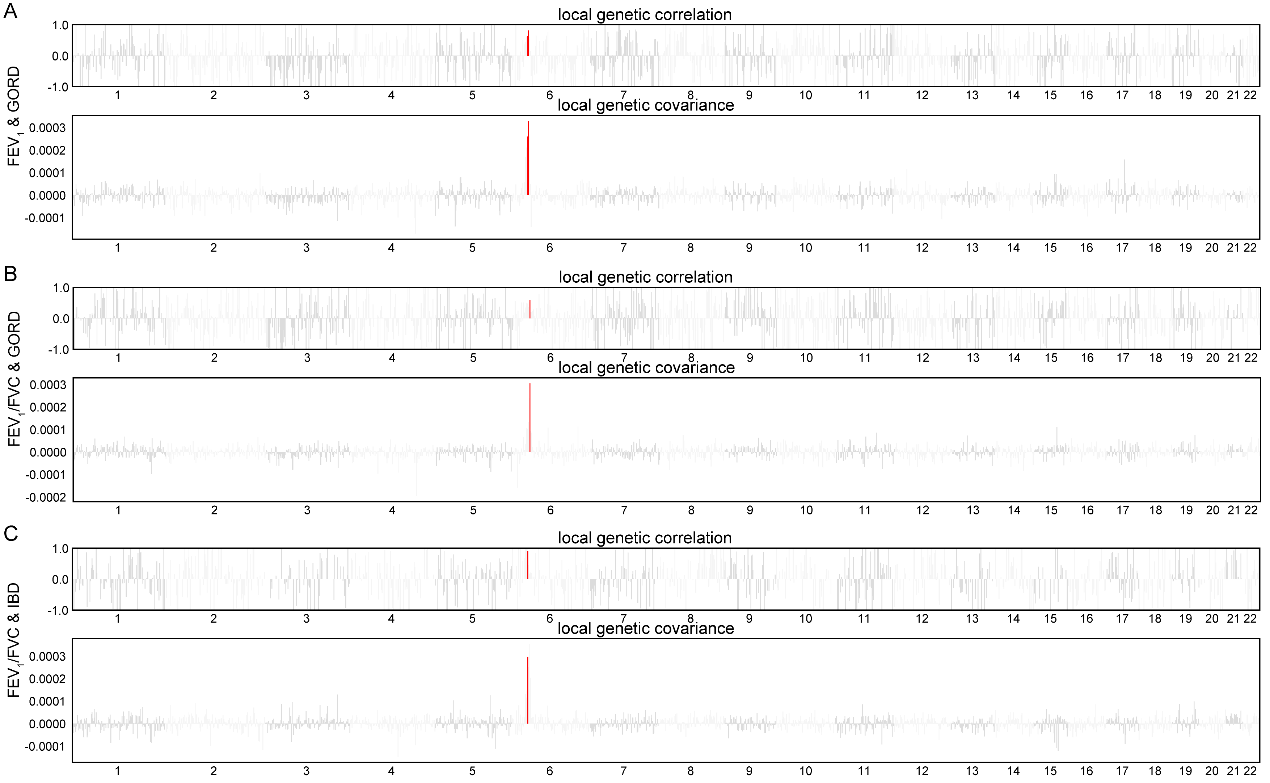


**Fig. S1 Local genetic correlations in pairwise traits**

(**A**) FEV_1_-GORD, (**B**) FEV_1_/FVC-GORD and (**C**) FEV_1_/FVC-IBD. The length of the bar represents the magnitude of local genetic correlation or covariance in one LD block region. Regions with significant local genetic correlation (*P*<0.05/1703) were colored red. A total of four regions located in MHC were identified.


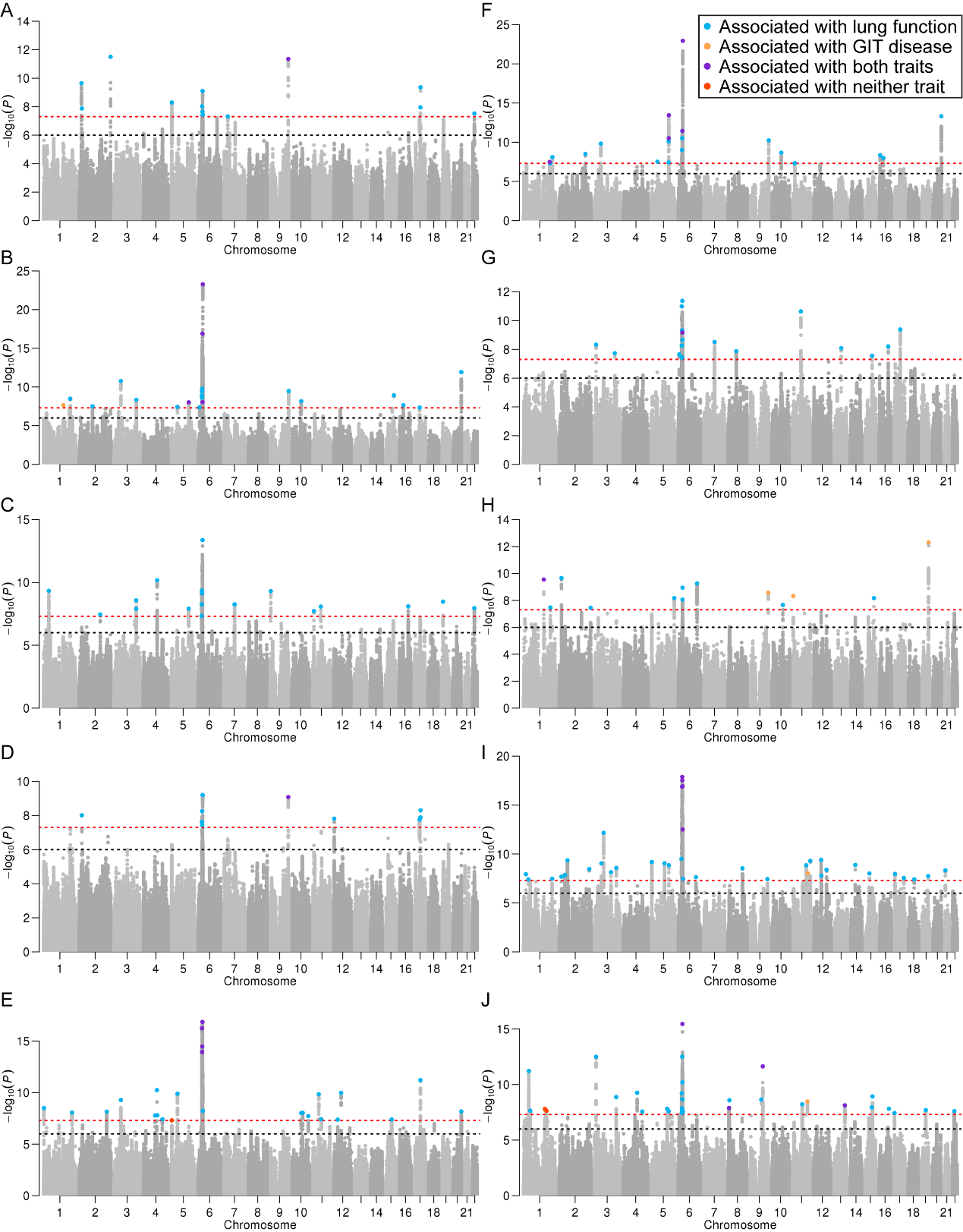


**Fig. S2 Manhattan plots of pairwise pleiotropic analyses**

**(A)** FEV_1_-PUD, **(B)** FEV_1_-IBD, **(C)** FEV_1_-IBS, **(D)** FVC-PUD, **(E)** FVC-GORD, **(F)** FVC-IBD, **(G)** FVC-IBS, **(H)** FEV_1_/FVC-PUD, **(I)** FEV_1_/FVC-GORD, and **(J)** FEV_1_/FVC-IBS. GIT, gastrointestinal tract. The red dashed lines indicate the genome-wide significance level at *P*=5×10^-8^, and the black dashed lines indicate the suggestive significance level at *P*=1×10^-6^. The blue point indicates the locus was associated with lung function; orange indicates the locus was associated with GIT disease; purple indicates the locus was associated with both traits; red indicates the locus was associated with neither trait.


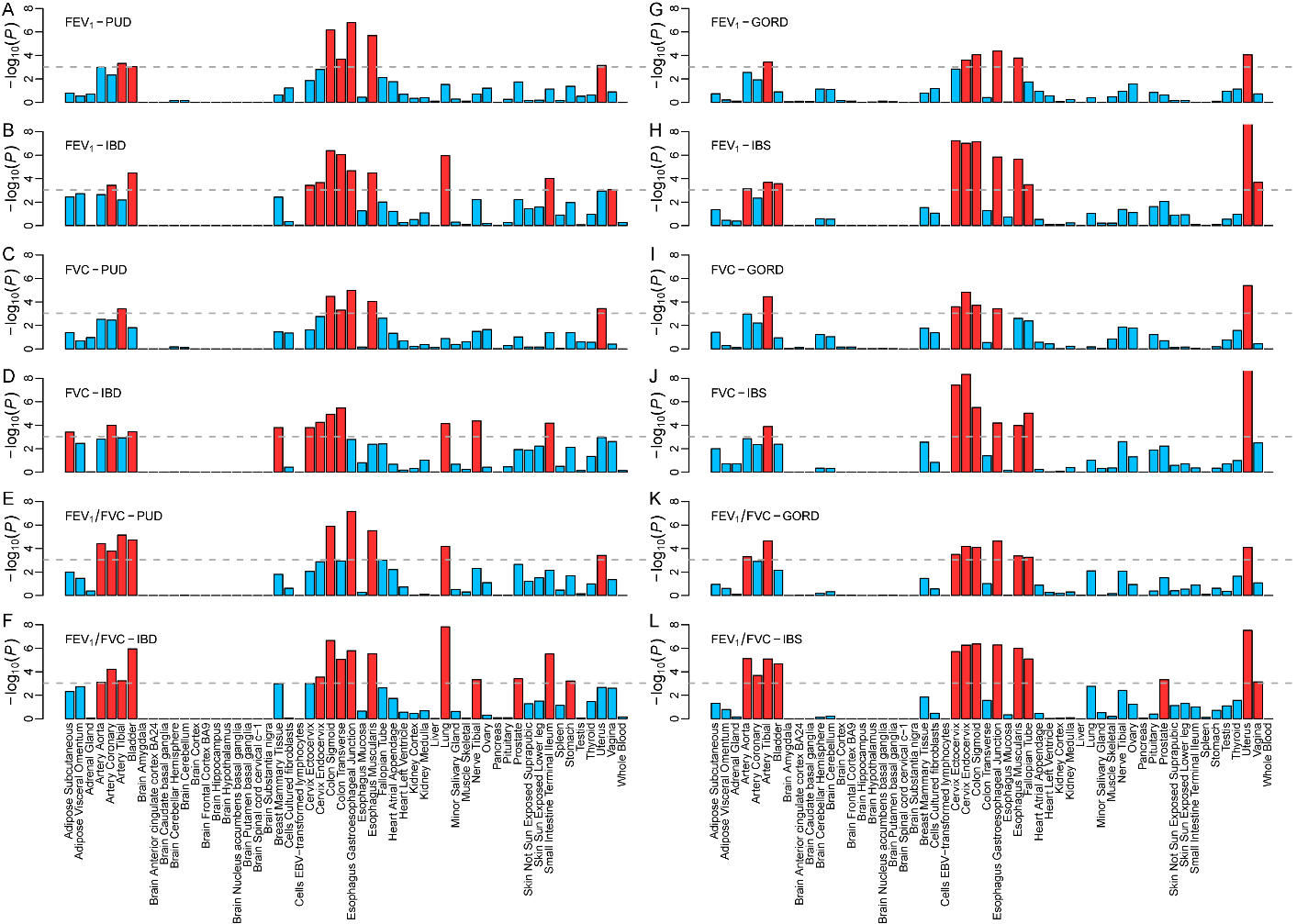


**Fig. S3 Relevant tissues identified by MAGMA**

A total of 54 tissues from the GTEx v8 were tested for associations with each trait pair by MAGMA gene property tests. The corresponding trait pair was marked on the top left. The gray dashed lines indicate the Bonferroni-corrected significance level at *P*=0.05/54=9.26×10^-4^. Red indicates significantly relevant tissues.


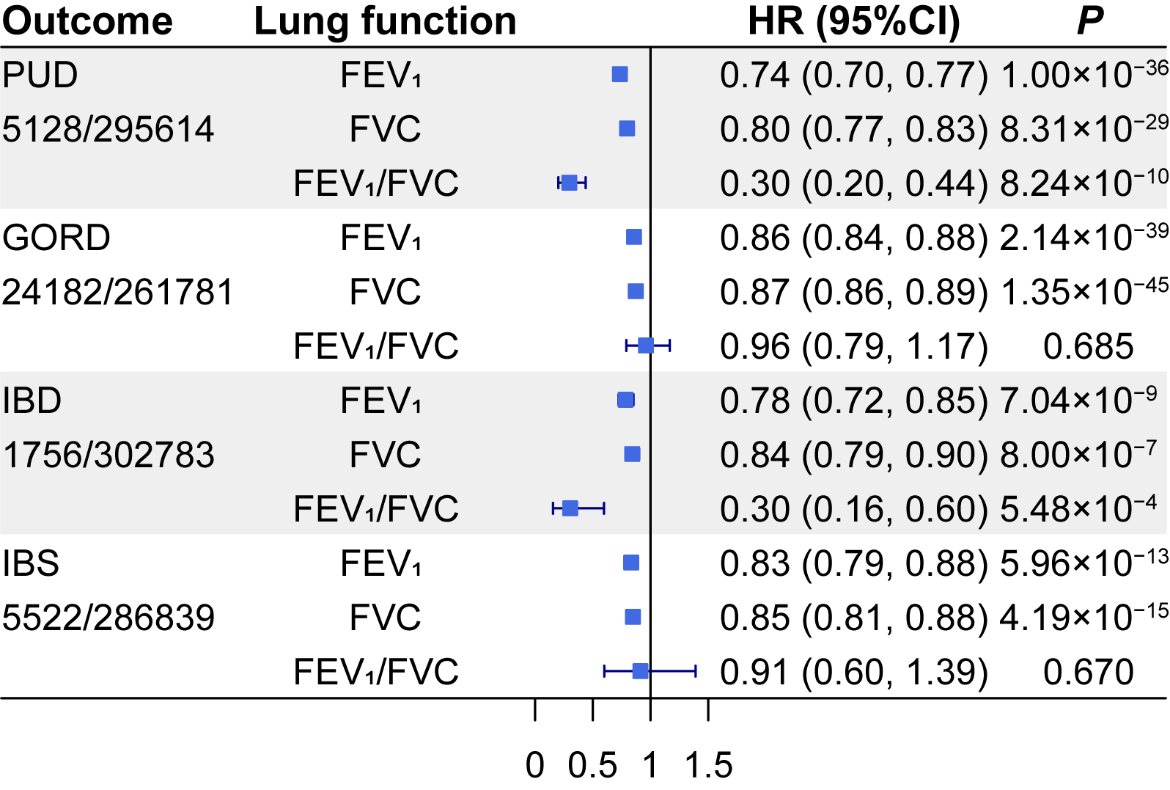


**Fig. S4 Cox proportional hazard models of lung function on gastrointestinal tract diseases**

The number of incident cases and controls for each disease were listed in the first column. HR, hazard ratio; CI, confidence interval.


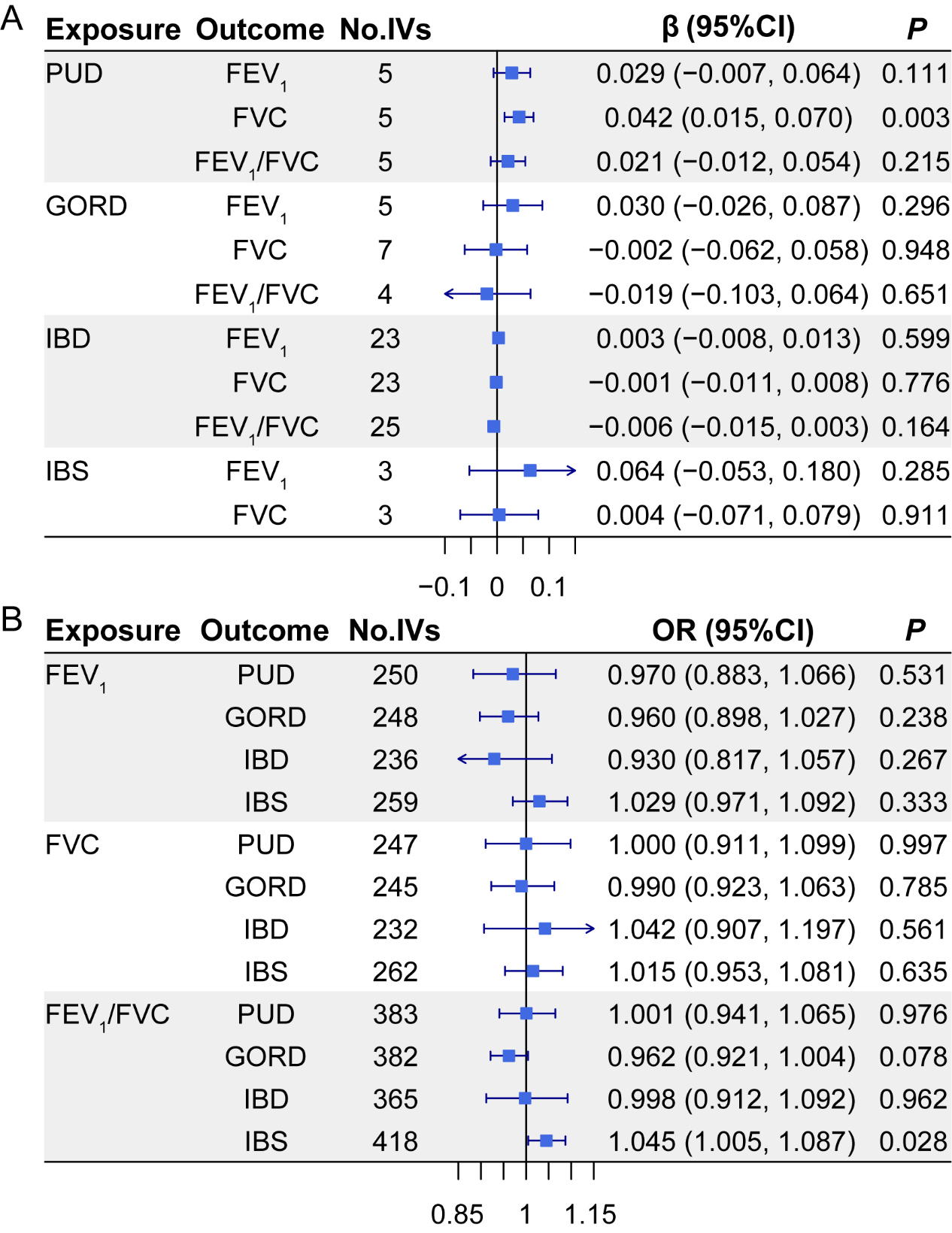


**Fig. S5 Mendelian randomization results between lung function and gastrointestinal tract diseases using the inverse-variance weighted method**

(**A**) Causal effects of gastrointestinal tract diseases on lung function. (**B**) Causal effects of lung function on gastrointestinal tract diseases. No.IVs is the number of instrumental variables; β (95% CI) and OR (95% CI) represent the effect size and odds ratio with the corresponding 95% confidence interval. No significant causal effect was detected after the Bonferroni correction (*P*<0.05/24=0.002).


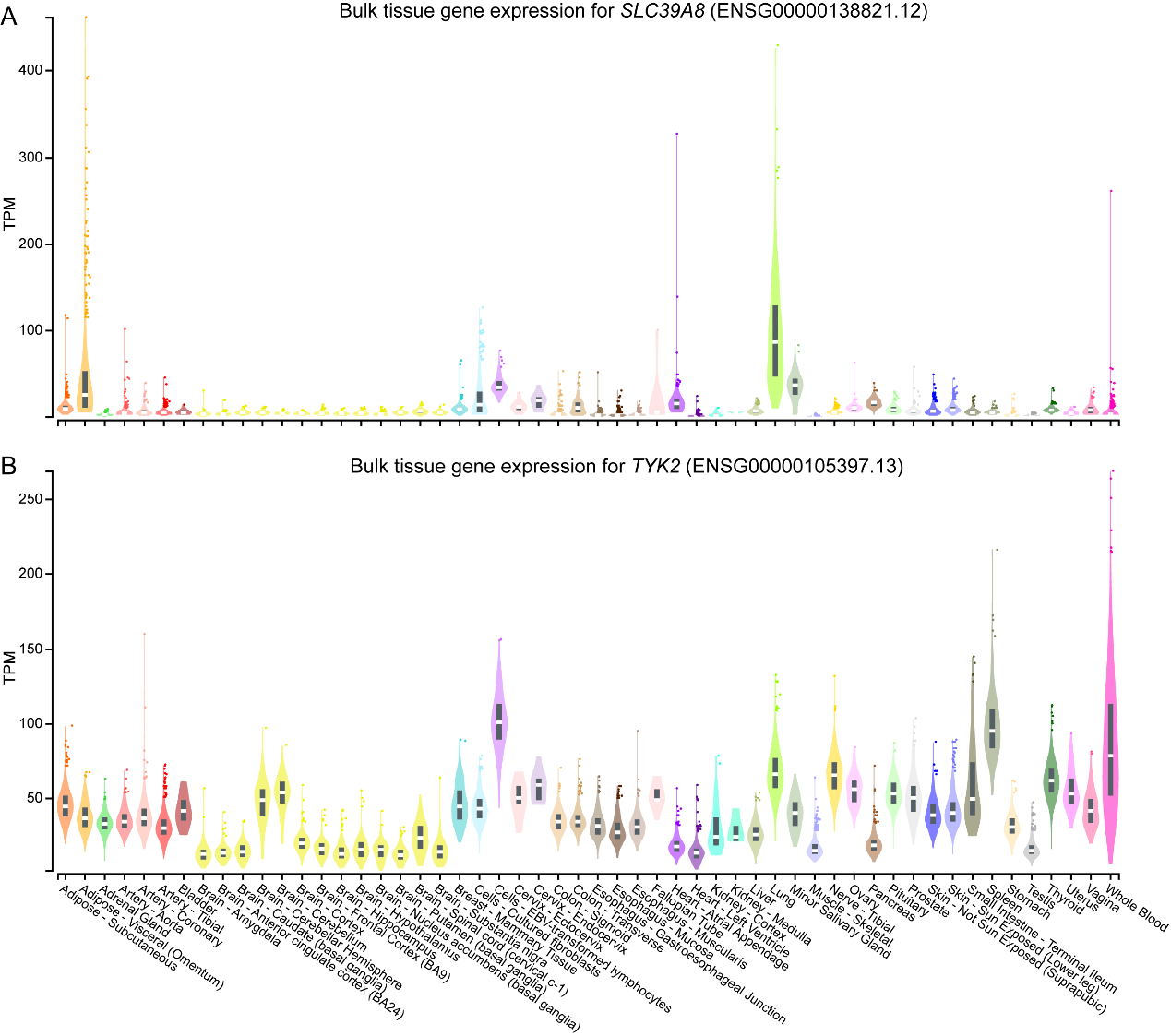


**Fig. S6 Gene expression in the GTEx v8 54 tissues**

**(A)** *SLC38A8* and **(B)** *TYK2*. *SLC38A8* and *TYK2* are both highly expressed in the lung. This figure was downloaded from the GTEx portal.
